# The Class I-Specific HDAC Inhibitor MS-275 Decreases Motivation to Consume Alcohol and Relapse in Heavy Drinking Rats

**DOI:** 10.1093/ijnp/pyv029

**Published:** 2015-04-23

**Authors:** Jerome Jeanblanc, Sandrine Lemoine, Virginie Jeanblanc, Stéphanie Alaux-Cantin, Mickaël Naassila

**Affiliations:** INSERM ERI 24, Groupe de Recherche sur l’Alcool et les Pharmacodépendances, Université de Picardie Jules Verne, Centre Universitaire de Recherche en Santé, Centre Hospitalo-Universitaire (CHU sud), Amiens, France (Drs J. Jeanblanc, Lemoine, Alaux-Cantin, and Naassila); Plateforme Animalerie du Pôle Santé – Université de Picardie Jules Verne, AmiensFrance (Ms V. Jeanblanc).

**Keywords:** Heavy drinking, epigenetic, alcohol addiction, treatment, relapse

## Abstract

**Background:**

New strategies for the treatment of alcohol dependence are a pressing need, and recent evidence suggests that targeting enzymes involved in epigenetic mechanisms seems to have great potential. Among these mechanisms, alteration of histone acetylation by histone deacetylases is of great importance for gene expression and has also been implicated in addiction. Here, we examined whether intra-cerebroventricular administration of MS-275, a class I-specific histone deacetylase inhibitor, could alter ethanol self-administration, motivation to consume ethanol, and relapse in heavy drinking rats.

**Methods:**

Male Long Evans rats trained to self-administer high levels of ethanol received intra-cerebroventricular micro-infusions of MS-275 (250 µM, 500 µM, and 1000 µM) 3 hours prior to the self-administration sessions.

**Results:**

First, we demonstrated that intra-cerebroventricular infusion of MS-275 increases acetylation of Histone 4 within the nucleus accumbens nucleus accumbens and the dorsolateral striatum. Second, we observed that MS-275 decreases ethanol self-administration by about 75%. We found that 2 consecutive daily injections are necessary to decrease ethanol self-administration. Additionally, the dose-response curve test indicated that MS-275 has a U-shape effect on ethanol self-administration with the dose of 500 µM as the most efficient dose. Furthermore, we showed that MS-275 also diminished the motivation to consume ethanol (25% decrease), and finally, we demonstrated that MS-275 reduced relapse (by about 50%) and postponed reacquisition even when the treatment was stopped.

**Conclusions:**

Our study confirms the potential therapeutic interest of targeting epigenetic mechanisms in excessive alcohol drinking and strengthens the interest of focusing on specific isoforms of histone deacetylases.

## Introduction

Alcohol use disorder is a devastating illness with a profound health impact, and its development is dependent on both genetic and environmental factors. This disease occurs over time and requires changes in brain gene expression. There is converging evidence suggesting that the epigenetic processes may play a role in the alcohol-induced gene regulations and behavior such as the intervention of DNA methylation and histone acetylation (Simon-O’Brien et al., 2014). Histone acetylation, like histone methylation, is a highly dynamic process regulated by 2 classes of enzyme: histone acetyltransferases and histone deacetylases (HDACs). To date, 18 human HDAC isoforms have been characterized, and based on their sequence homologies and cofactor dependencies, they have been phylogenetically categorized into 4 main classes: classes I, II (a and b), III, and IV. In the brain, expression of the different classes of HDACs varies between cell types and also in their subcellular localization (nucleus and/or cytosol) (Vilpoux et al., 2009). Furthermore, a recent study has shown that a single ethanol exposure inhibits HDAC activity and increases both H3 and H4 histone acetylation within the amygdala of rats (Alaux-Cantin et al., 2013).

In the brain of alcoholic patients, ethanol has been shown to induce histone-related and DNA methylation epigenetic changes in several reward regions involved in reward processes such as hippocampus, prefrontal cortex, and amygdala (Cleren et al., 1999; Vilpoux et al., 2002; Warnault et al., 2007). We recently demonstrated alteration of histone H3 acetylation levels in several brain regions from the reward circuit of rats made dependent to alcohol after chronic and intermittent exposure to ethanol vapor (Simon-O’Brien et al., 2014). In neuronal cell line culture, ethanol was shown to induce HDAC expression (Agudelo et al., 2011, 2012). In mouse and rat brain, numerous studies reported epigenetic alterations following ethanol exposure (Pandey et al., 2008; Pascual et al., 2012; Starkman et al., 2012; Warnault et al., 2013). We also demonstrated that both the expression of genes and the activity of enzymes involved in epigenetic mechanisms are changed after repeated administrations of ethanol in mice sensitized to the motor stimulant effect of ethanol (a model of drug-induced neuroplasticity) (Botia et al., 2012).

Numerous studies have shown that HDAC inhibitors are able to counter ethanol-induced behaviors and the ethanol-induced changes in the levels of HDAC and/or levels of acetylated HDAC. For example, trichostatin A (TSA) treatment caused the reversal of ethanol-induced tolerance, anxiety, and ethanol drinking by inhibiting HDAC activity, thereby increasing histone acetylation in the amygdala of rats (Alaux-Cantin et al., 2013; Sakharkar et al., 2014; You et al., 2014). Pandey and colleagues (2008) have shown that TSA prevented the development of ethanol withdrawal-induced anxiety in rats by rescuing deficits in histone acetylation induced by increased HDAC activity in the amygdala. We have demonstrated that treatment with the HDAC inhibitor sodium butyrate blocks both the development and the expression of ethanol-induced behavioral sensitization in mice (Legastelois et al., 2013). In this context, converging evidence indicates that HDAC inhibitors could be useful in counteracting ethanol-induced gene regulations via epigenetic mechanisms, that is, HDAC inhibitors could affect different acetylation sites and may also alter the expression of different genes that could in turn counteract the effect of ethanol. Recent work in rodents has shown that systemic administration of pan HDAC class I and II inhibitors, TSA and N-hydroxy-N′-phenyl-octanediamide [SuberoylAnilide Hydroxamic Acid] (SAHA), and of the more selective inhibitor (mainly HDAC1 and HDAC9) MS-275 decrease binge-like alcohol drinking in mice (Warnault et al., 2013). Warnault et al. (2013) also showed that SAHA selectively reduces ethanol operant self-administration and seeking in rats. Our previous study revealed that MS-275 strongly decreased operant ethanol self-administration in alcohol-dependent rats when administered 30 minutes before the session at the second day of i.p. injection (Simon-O’Brien et al., 2014). However, we do not have any data on both motivation and relapse, and we cannot exclude peripheral effect (secondary or motor effects) when the compound is administered via the intraperitoneal route.

In summary, numerous studies have revealed a potential interest of counteracting ethanol-induced epigenetic changes and also alcohol drinking behavior, but to our knowledge no data are available yet regarding the effect of selective HDAC inhibitor on motivation to self-administer ethanol and on relapse after protracted abstinence.

In the present study, we examined whether intra-cerebroventricular administration of the inhibitor MS-275 could alter not only ethanol self-administration but also both motivation to consume ethanol and ethanol relapse after protracted abstinence in high drinking rats. MS-275 is more specific than TSA or valproic acid, for example (Khan et al., 2008). MS-275 is often described as an HDAC1 inhibitor. Indeed, it has been suggested that it will mainly inhibit HDAC1 (EC_50_=181nM) but also HDAC9 (EC_50_=505nM) (Khan et al., 2008).

## Methods

### Animals

Male Long-Evans rats (320–345g at the beginning of the experiment) were obtained from Charles River (L’Arbresle, France). Animals were individually housed under a light/dark cycle of 12 hours (lights on at 7:00 am) with food and water available ad libitum. Experiments were carried out in accordance to the guidelines for Care and Use of Laboratory Animals (National Institutes of Health) and the European community regulations for animal use in research (CEE no. 86/609) and were approved by the local research ethics committee (CREMEAP; n° 260912-10).

### Reagents

MS-275 was purchased from Selleckchem (Munich, Germany) and dissolved to a concentration of 250 µM, 500 µM, and 1000 µM in a 10% DMSO (Sigma-Aldrich, Saint Quentin, France) 90% artificial cerebrospinal fluid solution (CMA Microdialysis, Solna, Sweden). The doses of MS-275 were chosen accordingly to its IC_50_ towards HDAC1 generally observed ( approximately 0.2–2 µM) (Khan et al., 2008; Nishioka et al., 2008) and to the volume of the brain cerebrospinal fluid (90 µL) (Pardridge, 2011). Ethanol 96% was purchased from VWR (Fontenay-sous-bois, France).

### Effects of Intra-Cerebro-Ventricular Infusion of MS-275 on Histone H4 Acetylation

MS-275 500 µM or MS-275 1000 µM was micro-infused twice within the lateral ventricle 24 hours apart with vehicle in deeply anesthetized (isoflurane) animals as described in detail in supplementary Methods. A total of 12 rats were used and assigned to the different groups. Two hours after the second micro-infusion, brains were collected after a transcardiac perfusion of paraformaldehyde (4%), postfixed overnight at 4°C, and soaked in 30% sucrose solution. Brains were sectioned (50 µm) using a vibratom (Leica VT1200S) in ice-cold phosphate buffer (for 1 L: 2.4g NaH_2_PO_4_, 11.36g Na_2_HPO_4_, pH 7.4). Floating sections were immunostained for Ac-H4K12 (Anti-acetyl-Histone H4 [Lys 12], rabbit monoclonal # 04-119, Millipore, Fontenay-sous-Bois, France) as described in supplementary Methods.

### Image Analysis

Immunolabeling was visualized using a light microscope connected to an image analysis system (Mercator, ExploraNova, LaRochelle, France). All images were acquired using the same light level by using a 10× objective lens and analyzed by a blind experimenter unaware of the experimental groups. A basal threshold was established and then applied to the images. Subsequently, the color digital image was classified into positive staining areas and background. Cells with immunoreactivity above this threshold were counted as Ac-H4K12 immuno-positive cells. Ac-H4K12 immuno-positive cells were counted in each of the brain regions studied and then normalized in areas of 100000 µm^2^. In addition, optical density (OD) was measured for each immuno-positive cell and then summed for each picture taken.

### Self-Administration of High Levels of Ethanol

To obtain large amounts of self-administered ethanol, we performed an induction of ethanol consumption in the home cage based on the 20% intermittent access protocol followed by several weeks of operant self-administration training as described in Jeanblanc et al. (2013) (for details, see supplementary Methods). This paradigm allows us to observe ethanol intake reaching a level near 1g/kg/30min. According to Carnicella et al. (2009), this level of ethanol consumption leads to a blood ethanol concentration around 60 mg%.

### Surgery

Stereotaxic surgery was used to implant each rat with a cannula into the lateral ventricle. For detailed description, see supplementary Methods.

### Intra-Cerebroventricular MS-275 Injections

MS-275 (250, 500, or 1000 µM) was micro-infused intra-cerebroventricular (i.c.v.) on 2 consecutive days 3 hours before the beginning of the operant self-administration sessions. Two microliters of each solution were micro-infused during a period of 2 minutes with an extra minute at the end of the infusion to allow diffusion of the solution. Micro-infusions were performed with a Harvard pump 11 Plus Advanced (Phymep, France) and 25-mL Hamilton syringes (1702N, Bonaduz, Switzerland). Injectors were built using stainless steel tubing (outer diameter: 0.229mm, inner diameter: 0.127mm, Phymep) and had a length of 13mm (1mm more than the tip of the guide cannula implanted in the rat brain). All the injections were performed following a Latin square design.

### Progressive Ratio Test

After rats were trained to self-administer ethanol (FR3-20% ethanol), a progressive ratio session was conducted 3 hours after the second injection of MS-275 500 µM (thus 27 hours after the first injection). In the progressive ratio session, the effort needed to get 1 reward (ie, number of presses on the active lever) was continuously increased after each reward delivery (3, 4, 5, 7, 9, 12, 15, 17, 20, 22, 25, 28, 30, 33, 35). The maximum effort rats were willing to make to obtain a single reward is represented by the breaking point and was evaluated as well as the total number of active lever presses during the 30-minute session.

### Extinction and Relapse to Alcohol Test

After 3 months of training in an operant self-administration chamber, all animals were subjected to extinction training, whereby presses on the active lever no longer produced any alcohol delivery. Extinction training sessions (30 minutes) were conducted for 13 consecutive days. Daily sessions continued until all rats reached an extinction criterion of <20% of its baseline response (number of lever presses by session) per session for 3 consecutive days.

### Intra-Cerebroventricular Microinjections of MS-275 and Reacquisition of Ethanol Operant Self-Administration

Operant self-administration behavior was extinguished in daily extinction sessions in which presses on the active lever were not followed by the delivery of the drug. Our criterion of extinction was 3 consecutive sessions with a level of active lever presses inferior to 20% of the baseline EtOH self-administration. Reacquisition was prompted by the presentation of a prime of EtOH (0.1mL of a 20% EtOH solution) immediately at the beginning of the session and was followed by the delivery of the drug after 3 consecutive active lever presses. Since 2 micro-infusions of MS-275 are necessary to observe an effect, we performed a first micro-infusion 3 hours before the last session of extinction and the second micro-infusion 3 hours before the first reacquisition session. To test whether the MS-275 effect continued as long as we micro-infused the treatment, we performed a third micro-infusion 3 hours prior to the second reacquisition session. Each of the micro-infusions was performed 24 hours apart.

After rats reached a stable baseline, a second period of extinction was performed (13 sessions), and a new reacquisition experiment occurred in which the injections (vehicle or MS-275) were counterbalanced to complete the groups in the Latin square design chosen for the experiments.

### Effect of MS-275 i.c.v. Micro-Infusions on Locomotor Coordination

As already described, rats were implanted with cannula aiming at the lateral ventricle. After a week of recovery and a week of training (see supplementary Methods), rats were micro-infused with the vehicle or MS-275 500 µM on 2 consecutive days and locomotor coordination was recorded 3 hours after each micro-infusion under an accelerating rotarod program (5–25 r.p.m. for 300 s). Latency to fall from the rod was recorded in seconds.

### Statistical Analysis

Data were analyzed by 1 or 2-way ANOVA with repeated measures, depending on the experiment, followed by a Tukey posthoc test when indicated by significant effects of treatments or interactions. For simple comparisons, data were analyzed by a Student’s *t* test. Significance for all tests was set at *P<*.05.

## Results

### MS-275 Micro-Infused into the Lateral Ventricle Increases Histone Acetylation in Different Brain Regions

**Figure 1.**
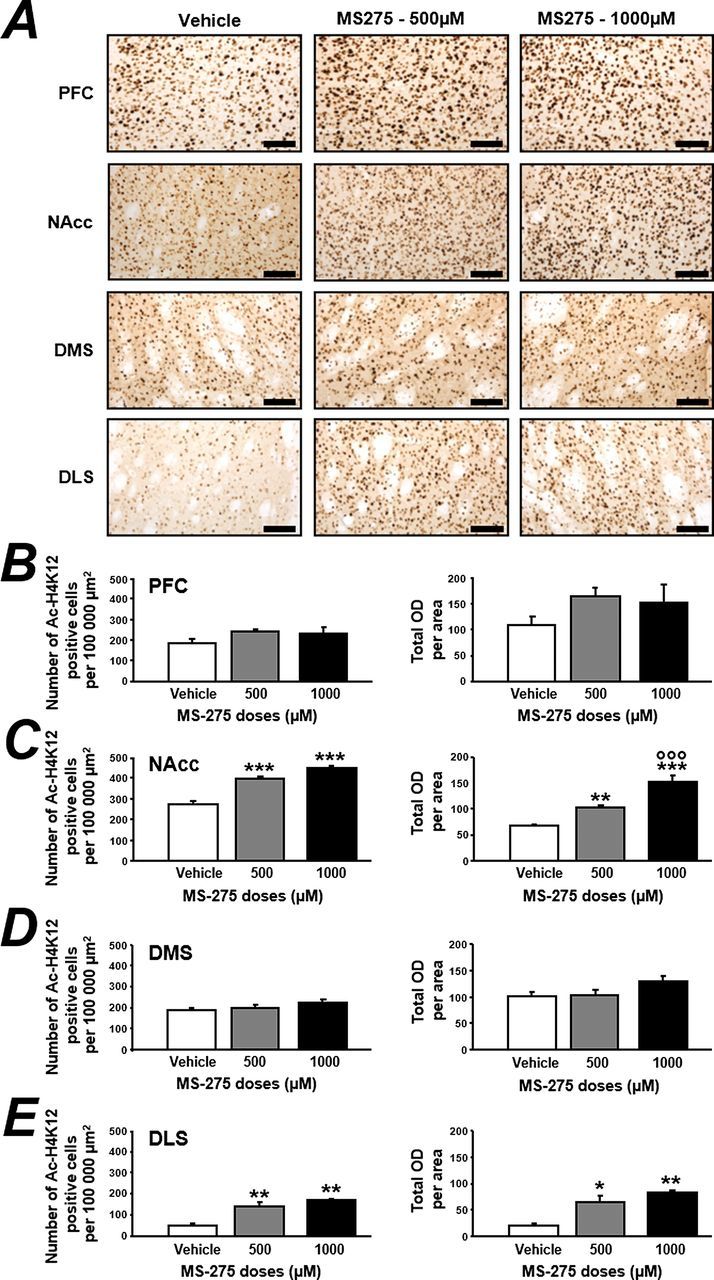
Intra-cerebroventricular (i.c.v.) micro-infusions of MS-275 differentially alter histone 4 acetylation depending on the brain regions. (A) MS-275 (0, 500, or 1000 µM) was micro-infused i.c.v. twice, 24 hours apart, and brains were collected 2 hours after the second injection. Histone 4 acetylation (Ac-H4K12) was revealed by immunohistochemistry in 4 different brain regions: prefrontal cortex (PFC), nucleus accumbens (NAcc), dorsomedial striatum (DMS), and dorsolateral striatum (DLS). The scale bar represents 100 µm. For each rat brain, a microphotography of the different structures of interest have been taken in both hemispheres. Both the number of positive Ac-H4K12 cells and the total optical density (OD) of the area were measured in the PFC (B), NAcc (C), DMS (D), and DLS (E). Results are expressed as mean ± SEM. Vehicle n=4, MS-275 500 µM n=5, MS-275 1000 µM n=3. *P<.05, **P<.01, ***P<.001 vs vehicle; °°°*P<.*001 vs MS-275 500 µM.

To ensure that 2 daily i.c.v. micro-infusions of MS-275 were able to modify histone acetylation in brain regions involved in reward and/or motivation, we performed an immunohistochemistry experiment specifically targeting H4K12 acetylation in forebrain structures (supplementary Figure S1) such as prefrontal cortex (PFC), the core part of the nucleus accumbens (NAcc), and the dorsomedial and dorsolateral striatum (DMS and DLS, respectively). We evaluated the number of Ac-H4K12 positive cells and the total OD of the area studied by microphotography after either micro-infusions of the vehicle, or the doses of 500 and 1000 µM of MS-275 (Figure 1a). An ANOVA analysis revealed that none of the MS-275 micro-infusions had an effect on ac-H4K12 levels within the PFC (Figure 1b) on the number of positive cells (F_(2,9)_=2.553, ns) or on the total OD (F_(2,9)_=2.368, ns). Within the NAcc (Figure 1c), the ANOVA revealed that the MS-275 treatment has a significant effect on the number of ac-H4K12 positive cells (F_(2,9)_=58.181, *P* < .001) and on the total OD (F_(2,9)_=40.526, *P* < .001). The posthoc analysis using a Tukey test on the number of ac-H4K12 positive cells indicated a significant difference between the vehicle group and the 500 and 1000 µM groups (all *P*<.001) but no difference between both MS-275 treatments (*P*=.051). Regarding the total OD, the Tukey test indicated a significant difference between the vehicle group and the doses of 500 (*P<.*01) and 1000 µM (*P<.*001) of MS-275 and between both doses of MS-275 (*P<.*001). Similarly to the PFC, MS-275 treatment had no effect on the number of Ac-H4K12 positive cells or on the total OD within the DMS (Figure 1d). The ANOVAs conducted on both the number of Ac-H4K12 positive cells (F_(2,9)_=1.355, ns) and the one conducted on the total OD (F_(2,9)_=1.938, ns) revealed no effect of the treatment. Finally, the ANOVA conducted on both the number of Ac-H4K12 positive cells (F_(2,9)_=17.703, *P<.*01) and on the total OD (F_(2,9)_=9.171, *P<.*01) within the DLS revealed a significant effect of the treatment (Figure 1e). The posthoc analysis on both the number of ac-H4K12 positive cells and on the total OD indicated a significant difference between the vehicle group and the 500 and 1000 µM groups (all *P* < .01) but no difference between both MS-275 treatments (all *P*>.05). Interestingly, no differences in the number of Ac-H4K12 immuno-positive cells were observed between the right and left NAcc or right and left DLS after MS-275 treatments (Supplemental Table 1).

### MS-275 Decreases Ethanol Operant Self-Administration

**Figure 2.**
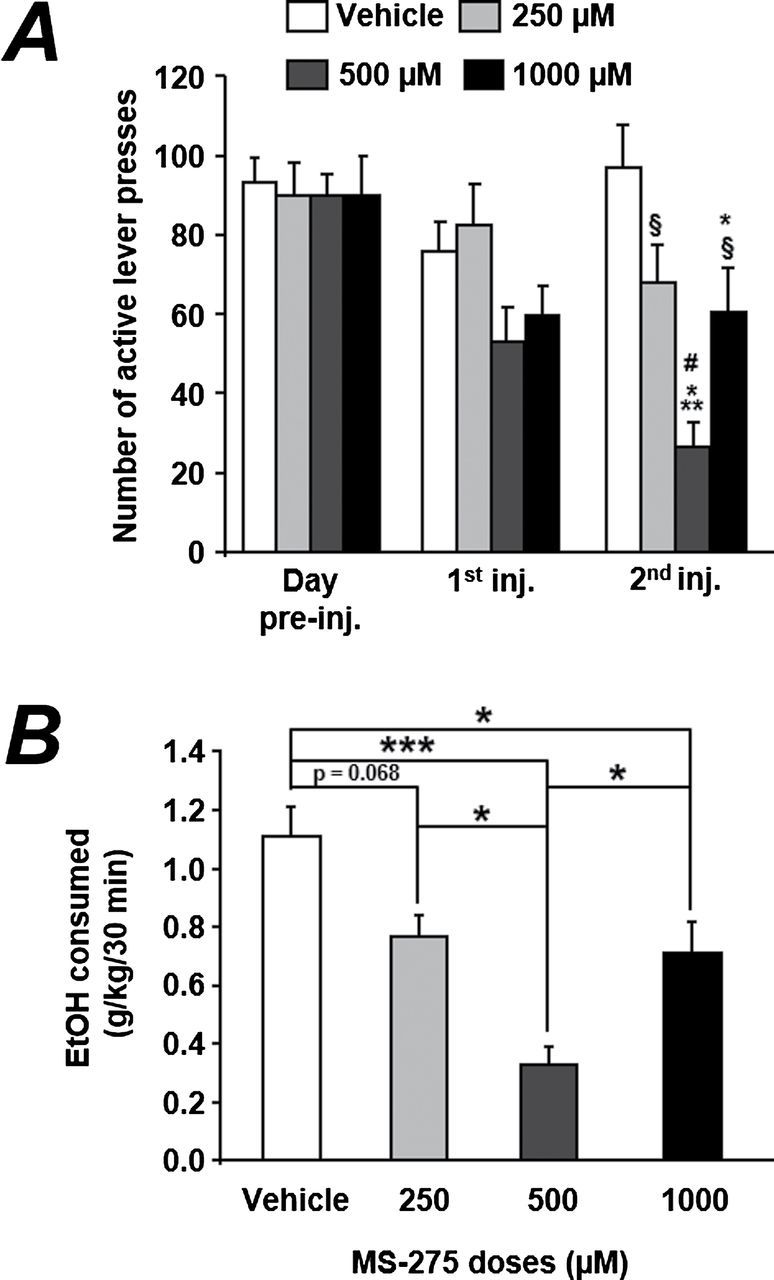
Dose-response curve of the MS-275 effect on EtOH self-administration. (A) The dose of 500 µM injected during 2 consecutive days is the most efficient dose of MS-275 on the decrease in lever presses on the active lever. Results are expressed as mean ± SEM. n=10. *P<.05, ***P<.001 vs vehicle second injection; #*P<.*05, ###*P<.*001 vs respective group day before; §*P<.*05 vs MS-275 500 µM dose within the same day. (B) EtOH consumed during the self-administration following the second injections of MS-275. Results are expressed as mean ± SEM EtOH consumed (g/kg/30min). n=10, *P<.05, ***P<.001.

First we evaluated the effect of 2 consecutive i.c.v. injections of MS-275 on the number of active lever presses during EtOH operant self-administration sessions. The injections were performed 3 hours prior to the beginning of the 30-minute operant self-administration sessions. As shown in Figure 2a, we observed that MS-275 decreased EtOH operant self-administration for the dose of 500 µM. A 2-way ANOVA with repeated measures indicated a main effect of the factors treatments (F_(3,72)_=5.48, *P<.*001) and time (F_(3,72)_=24.1, *P<.*001) and revealed an interaction between both factors (F_(9,72)_=3.11, *P<.*01). The posthoc analysis showed significant difference between the 500-µM dose and vehicle only after the second injection (*P<.*001). In addition, the dose of 1000 µM was significantly different from both vehicle and the dose of 500 µM (all *P*<.05). The level of number of presses was also significantly different within the 500 µM dose between the first and second injections. When focusing on the levels of EtOH consumed during this second injection day (Figure 2b), a 1-way ANOVA with repeated-measures analysis revealed a main effect of the factor treatment (F_(3,27)_=11.73, *P<.*001) and the posthoc test indicated significant differences between the vehicle group and the MS-275 500 µM and 1000 µM groups (*P<.*001 and *P<.*05, respectively). A trend toward a significant difference with the vehicle group was observed for the lowest dose of MS-275 (*P*=.068). The dose of 500 µM was also found to be different from the 2 other doses (all *P*<.05).

**Figure 3.**
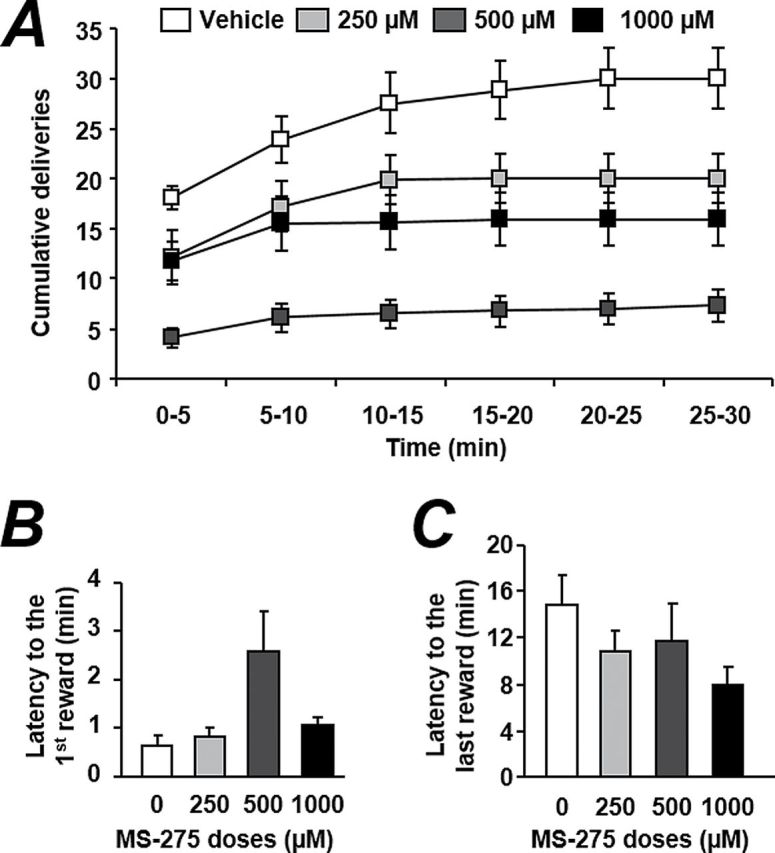
The second injection of MS-275 alters the pattern of EtOH self-administration. (A) Cumulative lever presses are differentially altered by MS-275 treatments. Results are expressed as mean cumulative lever presses. n=10. (B) Latency to obtain the first reward is not altered by the MS-275 treatments. Results are expressed as mean ± SEM of the latency to obtain the first reward (minutes). All rats not having at least one reward for any of the doses tested was removed from this analysis. n=7. (C) Latency to obtain the last reward of the 30-minute self-administration is not affected by the MS-275 treatments. Results are expressed as mean ± SEM of the latency to obtain the last reward (minutes). All rats not having at least one reward for any of the doses tested was removed from this analysis. n=7.

 The analysis of drinking pattern after the second injection of MS-275 was conducted using the cumulative deliveries and the latencies to obtain the first and last rewards. As shown in Figure 3a, the dose of 500 µM of MS-275 blocked the initiation of the drinking episode and flattened the cumulative curve. A 2-way ANOVA with repeated measures revealed a main effect of the factors treatment (F_(3,120)_=10.37, *P<.*001) and time (F_(5,120)_=40.43, *P<.*001) and indicated an interaction between both factors (F_(12,120)_=5.48, *P<.*001). The multiple comparison test pointed out significant differences between the group vehicle and the groups MS-275 500 µM (*P<.*001) and MS-275 1000 µM (*P<.*05).

We also found that the latencies to obtain the first and last rewards were not altered by the MS-275 treatments (Figure 3b-c).

### *MS-275* Decreases Motivation to Consume Ethanol

**Figure 4.**
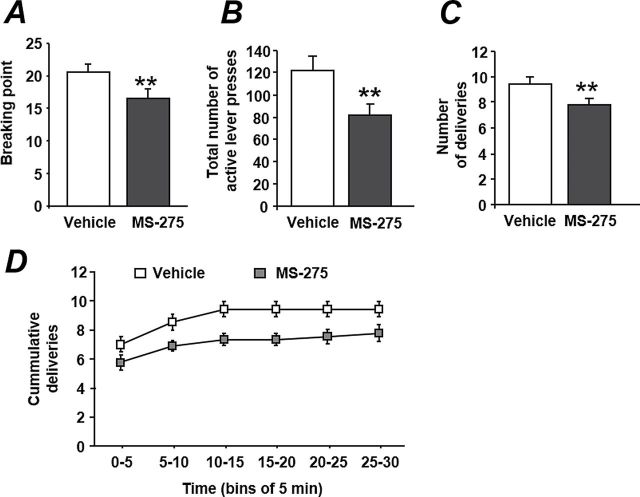
MS-275 decreases motivation to consume EtOH. The motivation to consume EtOH was evaluated during a progressive ratio experiment in which the breaking point (A), the total number of presses (B), and the number of deliveries (C) were recorded. (D) Cumulative deliveries during the progressive ratio experiment. All the results are expressed as mean ± SEM. n=9, **P<.01.

To evaluate the effect of MS-275 on the motivation to consume EtOH, we performed a progressive ratio experiment in which the effort to obtain 1 dose of EtOH increased after each delivery. We chose to test the 500-µM dose after 2 consecutive injections in this experiment. We found that the breaking point, the total number of active lever presses, and the number of deliveries were significantly decreased by the i.c.v. injection of MS-275 (Figure 4a-c, all *P*<.01). An analysis of the pattern of deliveries during this progressive ratio experiment revealed a main effect of the factors treatment (Figure 4d, F_(1,40)_=17.66, *P<.*001) and time (F_(5,40)_=26.5, *P<.*001) but no interaction between both factors (F_(5,40)_=1.52, ns). The posthoc analysis indicated that at all the time points, the MS-275 group was significantly different from the vehicle group (all *P<.*05).

### Reacquisition of Ethanol Operant Self-Administration

One of the major issues in the treatment of alcohol dependence is relapse after abstinence. Here, we extinguished the operant behavior during sessions in which the presses on the active lever did not produce any consequences: no light cue and no delivery of EtOH. This period of extinction lasted 13 days (supplementary Figure S2). Rats were then submitted to a reacquisition phase in which 3 consecutive presses on the active lever induced the occurrence of the light cue and the delivery of EtOH. Since the maximal effect of 500 µM MS-275 was observed after 2 consecutive days of injections, rats were first injected on the last day of extinction and then injected on the first day of reacquisition.

**Figure 5.**
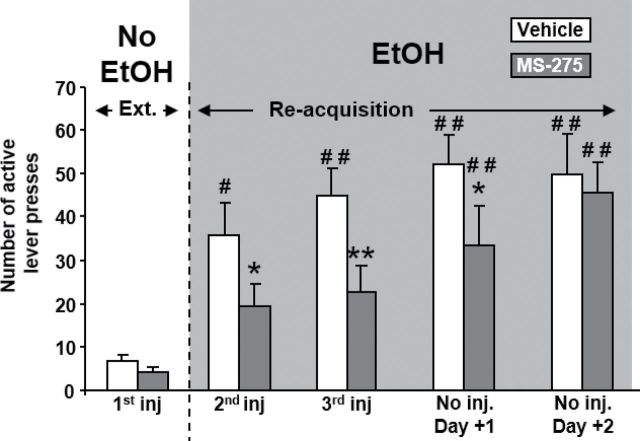
MS-275 postpones the relapse. After a period of extinction, rats were submitted to a reacquisition experiment in which both light cue and EtOH were again available during the sessions. MS-275 was micro-injected during 3 consecutive days with the first injection occurring on the last day of extinction. n=12, *P<.05 and **P<.01 vs vehicle within same day of injection; #*P<.*01 and ##*P<.*001 vs last day of extinction within same treatment.

The number of active lever presses was recorded during 3 days of injection and 2 days following the last injection of MS-275 or its vehicle. First, we observed that MS-275 did not induce relapse by itself when injected on the last day of extinction. We also found that MS-275 blocked the relapse as it was injected but had no further effect when it was not administered (Figure 5). A 2-way ANOVA with repeated measures conducted on the reacquisition data revealed a main effect of the factors treatment (F_(1,44)_=14.13, *P<.*01) and time (F_(4,44)_=17.73, *P<.*001) but no interaction between both factors (F_(4,44)_=1.42, ns). The posthoc analysis pointed out that the vehicle group relapsed after the first session of reacquisition (*P<.*01 vs vehicle group last day of extinction) and reached a new stable baseline level after the second and third reacquisition sessions (*P<.*001 vs vehicle group last day of extinction). In contrast, the MS-275 group did not relapse until the end of the MS-275 injections. Indeed, the multiple-comparison test did not reveal any differences within the MS-275 group between injections 1, 2, and 3. On the first day with no injection, the MS-275 group relapsed (*P<.*01 vs MS-275 group last day of extinction) and reached the levels of the vehicle group on the second day with no injection. No significant differences between both vehicle and MS-275 groups were detected on the last day of extinction, whereas the groups significantly differed on the 3 first reacquisition sessions (first: *P<.*05; second: *P<.*01; and third: *P<.*05).

**Figure 6.**
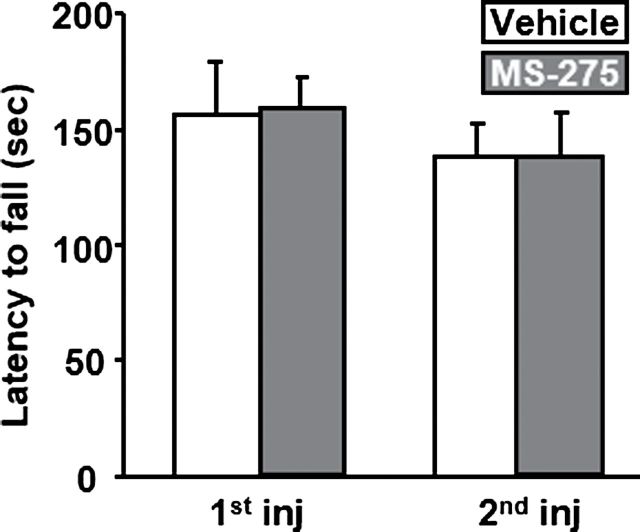
MS-275 does not alter locomotor activity. Rats trained to stay on a rotarod were submitted to 2 test sessions under an accelerating schedule from 5 to 25 r.p.m. for 5 minutes at 3 hours after 1 or 2 micro-infusions of a dose of 500 µM of MS-275. Results are expressed as mean ± SEM latency to fall from the rod. n=7.

As a control for locomotor activity, we performed an accelerating rotarod experiment after 1 and 2 i.c.v. micro-infusions of either the vehicle or the 500 µM dose of MS-275. The latency to fall from the rod was recorded 3 hours after each micro-infusion and is depicted in Figure 6. The 2-way ANOVA with repeated measures conducted on the data obtained revealed no effect of the factors time (F_(1,6)_=0.593, ns) and treatment (F_(1,6)_=0.023, ns) and also indicated no interaction between both factors (F_(1,6)_=0.027, ns).

To ensure that the effect of MS-275 on the second day was not a prolonged effect of the first injection coupled with an effect of the second injection, we performed a single injection. We found that, despite a trend, the single injection did not decrease EtOH operant self-administration on the day of injection and had no more effect on the following day (supplementary Figure S3).

## Discussion

We have previously shown that i.p. injection of the pan HDAC inhibitor sodium butyrate suppresses 10% ethanol operant self-administration in ethanol-dependent animals (Simon-O’Brien et al., 2014). We also confirmed in a preliminary experiment that the effect of sodium butyrate is mimicked (both efficacy and delay of action) by the i.c.v. injection of MS-275, thus indicating that the effect of the pan inhibitor is mediated by targeting HDAC and not to other unknown nonspecific effects (Simon-O’Brien et al., 2014). The preliminary results also opened new perspectives in terms of exploring the interest of targeting specific class of HDAC.

In this study, we choose to directly micro-infuse MS-275 into the brain (i.c.v.) and not i.p. (Warnault et al., 2013; Simon-O’Brien et al., 2014) to avoid a possible systemic effect of MS-275, since only 10 to 15% of MS-275 is distributed to the brain after i.p. injection at a dose of 40mg/kg in mice (Hooker et al., 2010). First, we demonstrated that i.c.v. micro-infusions of MS-275 increase acetylation of Histone 4 (Ac-H4K12) in some brain regions involved in reward/addiction but not in all, as already demonstrated with sodium butyrate (Simon-O’Brien et al., 2014). Indeed, a strong increase of Ac-H4K12 has been observed in the core part of the NAcc and in the lateral part of the dorsal striatum, while no effect of MS-275 was seen in the prelimbic area of the prefrontal cortex or in the dorsomedial striatum. This result is particularly interesting, since one can expect that epigenetic changes will be observed in specific brain regions and thus that depending on the specificity of the HDAC inhibitor it may have particular behavioral outcome.

The current report shows that the HDAC1 inhibitor MS-275 dose-dependently suppressed 20% ethanol operant self-administration in high drinking rats (rats drinking around 1.1g pure ethanol/kg/30min). More precisely, the U-shaped dose-response analysis revealed a hormetic response. The intermediate dose of MS-275 reduced very efficiently (75% decrease) ethanol intake at the second day of injection, while the two other doses displayed only limited effect. MS-275 was micro-infused within the lateral ventricle on 2 consecutive days and 3 hours prior to the operant self-administration session. We confirmed that one injection is not efficient to decrease ethanol operant self-administration when measured a day after and thus that the effect observed is a cumulative effect of both injections and not a postponed effect of the first injection, which would happen the day after. The delay of 3 hours between the micro-injection and the behavioral test was chosen to give time to the HDAC1 inhibitor to act on its target and to produce its final effect in modifying protein expression. The strong effect of the 500-µM dose, even micro-injected 3 hours prior to the test, suggests that some epigenetic modifications can occur and may be responsible for the effect observed. Moreover, since the MS-275 half-life is approximately 1 hour in rats (Ryan et al., 2005), the changes in behavior observed cannot be due to the presence of the drug anymore but to mid/long-term modifications occurring within the cells/nucleus. Both the delayed effect of MS-275 and the limited effect of the highest dose suggest that the effect on ethanol intake may involve changes in gene expression and thus that the behavioral outcome may not be linear but may be dependent upon several parameters such as the dose of the inhibitor and also the dose of ethanol. These results are also in line with our previous data showing that sodium butyrate prevented 1.0g/kg ethanol-induced sensitization but not the one at 2.0g/kg ethanol (Legastelois et al., 2013). At the 1.0-g/kg dose of ethanol used for sensitization, sodium butyrate prevented specific changes in gene expression and protein level such as the one of BDNF (Legastelois et al., 2013).

Analysis of the pattern of drinking indicated that the efficient dose of MS-275 decreased ethanol operant self-administration at the very beginning of the session and all along the session. Indeed, the initiation of the drinking episode was postponed (2.5 vs 0.6 minutes for the vehicle), and then no increase in ethanol consumption was observed for this particular dose during the session. In addition, the present data demonstrate, to our knowledge for the first time, that MS-275 reduces motivation to self-administer ethanol observed through the progressive ratio experiment showing a significant decrease (about 25%) in the breaking point value. The importance of our results lies in the fact that the motivational symptoms are key factors in alcohol consumption.

Another crucial addiction symptom is relapse, and thus a good medication may be effective not only on motivational aspects but also on craving and relapse. Our data demonstrated that the second injection of MS-275 reduced relapse (50% decrease) induced by ethanol priming and that this effect does last as long as the treatment is administered and even on the day after. It is noteworthy that MS-275 treatment does not induce relapse by itself, since it has no effect on the first day of injection and as it could be expected when one considers that ethanol is also inhibiting HDAC and may thus suggest a substitutive effect between ethanol and HDAC inhibitor.

Finally, we demonstrated that MS-275 micro-infused i.c.v. does not alter locomotor activity/coordination in an accelerating rotarod test, suggesting that the behavioral changes observed in all the different operant self-administration experiments are not due to deficit in locomotion.

In sum, all our data highlight that targeting a specific class of HDAC is effective in reducing excessive ethanol intake, motivation for ethanol drinking, and relapse after protracted abstinence. The effect of HDAC inhibitor may not be linked to a general effect on motivation and reward since we and others have shown that HDAC inhibitors have no effects on sucrose or saccharin operant self-administration (Warnault et al., 2013; Simon-O’Brien et al., 2014).

Ethanol affects virtually every neurotransmitter system in the central nervous system (reviewed in Vengeliene et al., 2008). Thus, the hypothesis of targeting HDAC enzymes and chromatin remodeling in order to modulate gene expression of multiple targets seems plausible. One caveat to the current data and their interpretation is that we only examined behavioral data and not the potential effect of MS-275 on gene and epigenetic regulations induced by ethanol intake, but this would require further set of experiments. Nevertheless, our data highlight the important role that specific HDAC isoforms may play in alcohol operant self-administration and relapse. As mentioned above, MS-275 will mainly inhibit HDAC1 and HDAC9; however, HDAC9 is 4 to 5 times less expressed than HDAC1 in the brain (Harrison and Dexter, 2013). Thus, by using i.c.v. micro-injections of MS-275, we probably observed an effect of MS-275 through its action on HDAC1.

HDAC inhibitors enhance gene expression making promoters accessible for transcriptional machinery. It is possible to argue that MS-275 treatment can increase expression of genes that may in turn downregulate ethanol intake such as BDNF, for example (Jeanblanc et al., 2009) and/or can counter gene regulations induced by ethanol. Indeed, it is already known that HDAC1 can form a complex with the methyl CpG binding protein 2 (MeCP2), which is a gene transcription repressor (Martinowich et al., 2003). This complex is able to bind the promoter IV of the *bdnf* gene silencing its expression (Martinowich et al., 2003). Thus, it is possible to suggest that inhibition of HDAC1 will prevent the complex formation and thus promote the expression of BNDF. In turn, BDNF will trigger the expression of effector genes such as the dopamine D3 receptor or dynorphin to reduce ethanol consumption (Jeanblanc et al., 2006; Logrip et al., 2008). In this regard, we have already shown that the HDAC inhibitor sodium butyrate may prevent ethanol-induced sensitization in part by preventing ethanol-induced change in *bdnf* expression and BDNF protein (Legastelois et al., 2013). It is noteworthy that blocking a specific class of HDAC may have also an impact on other classes of HDAC. For instance, MS-275 treatment leads to a decreased expression of HDAC8 in the NAcc (Covington et al., 2011). Interestingly, MS-275 has been shown to block the effects of cocaine by interacting with HDAC1 and molecular targets that regulate GABAA subunits gene expression (Kennedy et al., 2013).

Our data presented here are in line with the reduction of ethanol-induced behaviors by HDAC inhibitors and are the first to demonstrate that the specific inhibition of HDAC1 is also efficient and support the notion that alcohol addiction may involve epigenetic modifications. Identifying these epigenetic changes in the brain will be critical in both understanding the underlying etiology of alcohol addiction and in proposing novel therapeutic interventions.

## Statement of Interest

None.

## Supplementary Material

supplementary Figure S1
