# Supplementary material for: The Class I-Specific HDAC Inhibitor MS-275 Decreases Motivation to Consume Alcohol and Relapse in Heavy Drinking Rats
Source: Int J Neuropsychopharmacol. 2015 Apr 23;18(9):pyv029. doi: 10.1093/ijnp/pyv029 (PMC4576514; doi:10.1093/ijnp/pyv029)
Supplement: supplementary Figure S1 [file ijnp_pyv029_index.html]

Supplementary Data | International Journal of Neuropsychopharmacology

## Supplementary Data

Data files

**Files in this Data Supplement:**

- Supplementary Data - Supplementary Data
- Supplementary Data - Supplementary Data
- Supplementary Data - Supplementary Data
- Supplementary Data - Supplementary Data
