# Supplementary material for: The Class I-Specific HDAC Inhibitor MS-275 Decreases Motivation to Consume Alcohol and Relapse in Heavy Drinking Rats
Source: Int J Neuropsychopharmacol. 2015 Apr 23;18(9):pyv029. doi: 10.1093/ijnp/pyv029 (PMC4576514; doi:10.1093/ijnp/pyv029)
Supplement: supplementary Figure S1 [file pyv029Supplementary_Methods.docx]

**Supplementary Methods**

**Effects of i.c.v. Infusion of MS-275 on Histone H4 Acetylation**

Long Evans rats (350 ± 30 g) were anesthetized by inhalation of isoflurane (5% for the induction then 2.5-3% during surgery) and placed on a stereotaxic frame (David Kopf, Tujunga, CA). First the skin was removed and the skull cleaned (H_2_O_2_ 15%) and then a hole was drilled to allow the insertion of an injection cannula into the right lateral ventricule (antero-posterior coordinates: -0.8 mm from Bregma, +1.4 mm lateral from the midline, -3.0 mm ventral from skull surface). Twelve rats were distributed into 3 groups: 4 in the aCSF group, 5 in the MS-275 500 µM group and the 3 remaining rats were assigned to the MS-275 1000 µM group. Two microliters of the solutions (aCSF, MS-275 500 µM and MS-275 1000µM) were micro-infused at a speed of 1 µL/min. The injection cannula was slowly removed after an extra minute into the ventricule to allow the solutions to diffuse. The wound was closed using a cyanolicrate glue (Vetbound, 3M) and rats were placed back in their home cage for 24 hours. The following day, rats received a second micro-infusion of the same solution following the same procedure. Rats were then deeply anesthetized 2 hours after the second micro-infusion sodium pentobarbital (100 mg/kg) and perfused intracardially with ice-cold solutions of 0.9% NaCl and 4% paraformaldehyde. Brains were removed, post-fixed overnight at 4°C, and soaked in 30% sucrose solution. Brains were sectioned (50 µm) using a vibratom (Leica VT1200S) in ice-cold phosphate buffer (2.4 g NaH_2_PO_4_, 11.36 g Na_2_HPO_4_, pH 7.4).

Floating sections were immunostained for Ac-H4K12 (Anti-acetyl-Histone H4 [Lys 12], rabbit monoclonal # 04-119, Millipore, Fontenay-sous-Bois, France) as follows: slides were rinsed twice 10 minutes in PB 0.1 M then incubated for 30 minutes with 0.3% hydrogen peroxide in PB 0.1 M to quench endogenous peroxidases. Slices were rinsed again 3 times 10 minutes and then incubated overnight with the anti-ac-H4K12 antibody diluted (1:7500) in the blocking solution (PB 0.1 M, 0.1% BSA, 0.2% Triton X-100, 2% serum, pH 7.4). Slides were rinsed 4 times 10 minutes in PB and incubated for 2 hours with the biotinylated secondary antibody (1:2000, Goat anti-rabbit IgG, Jackson ImmunoResearch Laboratory, Suffolk, UK). Slices were then processed by using the Vectastain ABC Elite kit (Vector Laboratories, Burlingame, CA) and then stained with diaminobenzidine and H_2_O_2_ for 4 minutes.

**Surgery**

Stereotaxic surgery was used to implant each rat with a cannula into the lateral ventricle. Rats were continuously anesthetised with isoflurane during the surgery. Four holes were drilled for screws, and one other hole was drilled for the placement of cannulae (lateral ventricle: single cannula (26GA, 12 mm, Phymep). The coordinates for the lateral ventricle were +1.4 mm lateral to the medial suture, -0.8 mm posterior to bregma, and -3 mm from the skull surface. All the cannulae were implanted aiming at the right lateral ventricule. The cannulae were fixed with dental cement. Subject weights were monitored daily after the surgery to ensure recovery. One week after recovery, subjects returned to self-administration training and were habituated to the microinjection procedure with 2 sham injections. The experimental micro-injections began upon acquisition of stable responding for ethanol (10 sessions after the recovery from the surgery). The injectors used for each group extended 1 mm below the tip of the cannula.

**Self-Administration of High Levels of Ethanol**

To obtain large amounts of self-administered ethanol, we performed an induction of ethanol consumption in the home cage based on the 20% intermittent access protocol ([Jeanblanc et al., 2013](#_ENREF_3)). Naïve rats were exposed to a 20% intermittent access paradigm with access to 2 bottles, one containing tap water and the other a 20% ethanol solution, every other day in the homecage. This procedure induces escalation of ethanol consumption in several strains of rats ([Wise, 1973](#_ENREF_5); [Carnicella et al., 2009](#_ENREF_2); [Bito-Onon et al., 2011](#_ENREF_1)). After 4 to 5 weeks of 20% intermittent access, rats were trained to self-administer ethanol. The self-administration chambers contained 2 levers: an active lever for which presses resulted in delivery of a 0.1 mL fluid reward (a 20% ethanol solution), and an inactive lever, for which presses were counted, but no programmed events occurred. After 3 days under a fixed ratio 1 (FR1, 1 press delivers 1 reward) schedule, the rats were trained on an FR3 schedule (3 presses are required to receive 1 reward) during daily 30-minute sessions 5 days per week. Animals were trained for at least 5 weeks before the beginning of the experimental manipulations. The animals exhibiting <40 presses on the active lever at the end of the 5 weeks of training for 5 consecutive sessions were excluded from the study because of their low ethanol consumption ([Jeanblanc et al., 2014](#_ENREF_4)). During the self-administration sessions, number of lever presses and number of ethanol deliveries were recorded using PackWin software (Bioseb, Vitrolles, France). This paradigm allows us to observe ethanol intake reaching a level near 1 g/kg/30 min. According to Carnicella et al. (2009), this level of ethanol consumption leads to blood ethanol concentration around 60 mg%

**Effect of MS-275 i.c.v. Micro-Infusions on Locomotor Coordination**

Rats were implanted with cannula aiming at the lateral ventricule as described above. After a week of recovery, the rotarod training started. Rats were pretrained on a 5-lane rotarod (IITC Life Science, Woodland Hills, CA) and subsequently tested to establish individual baseline levels of performance. Pretraining was completed by placing the rat on the moving rotarod at 5 r.p.m. until they could perform at this speed for 60 seconds. Once rats reached this goal, the speed was increased across the sessions (10, 15, and 20 r.p.m.) during 2 daily sessions for 4 days. Rats were then micro-infused with aCSF or MS-275 500 µM on 2 consecutive days and locomotor coordination was recorded 3 hours after each micro-infusion under an accelearting rotarod program (5-25 r.p.m. for 300 seconds). The micro-infusions were counterbalanced the following week; thus, each rat was its own control.

**REFERENCES**

Bito-Onon JJ, Simms JA, Chatterjee S, Holgate J, Bartlett SE (2011) Varenicline, a partial agonist at neuronal nicotinic acetylcholine receptors, reduces nicotine-induced increases in 20% ethanol operant self-administration in Sprague-Dawley rats. Addict Biol 16:440-449.

Carnicella S, Amamoto R, Ron D (2009) Excessive alcohol consumption is blocked by glial cell line-derived neurotrophic factor. Alcohol 43:35-43.

Jeanblanc J, Coune F, Botia B, Naassila M (2013) Brain-derived neurotrophic factor mediates the suppression of alcohol self-administration by memantine. Addict Biol.

Jeanblanc J, Coune F, Botia B, Naassila M (2014) Brain-derived neurotrophic factor mediates the suppression of alcohol self-administration by memantine. Addict Biol 19:758-769.

Wise RA (1973) Voluntary ethanol intake in rats following exposure to ethanol on various schedules. Psychopharmacologia 29:203-210.
