# Supplementary material for: The Class I-Specific HDAC Inhibitor MS-275 Decreases Motivation to Consume Alcohol and Relapse in Heavy Drinking Rats
Source: Int J Neuropsychopharmacol. 2015 Apr 23;18(9):pyv029. doi: 10.1093/ijnp/pyv029 (PMC4576514; doi:10.1093/ijnp/pyv029)
Supplement: supplementary Figure S1 [file Supplemental_Table.docx]

**Supplemental Table 1**

| **Number of**  **Ac-H4K12 immune-positive cells** | **Nucleus accumbens core** | | **Dorsolateral striatum** | |
| --- | --- | --- | --- | --- |
|  | **Ipsilateral** | **Contralateral** | **Ipsilateral** | **Contralateral** |
| **aCSF** | 295.50 ± 9.3 | 257.00 ± 26.81 | 83.16 ± 24.58 | 79.16 ± 18.97 |
| **MS-275 500 µM** | 405.60 ± 14.69 | 393.87 ± 13.26 | 208.13 ± 24.26 | 234.13 ± 43.38 |
| **MS-275 1000 µM** | 460.22 ± 16.24 | 430.67 ± 29.06 | 270.67 ± 44.67 | 269.33 ± 23.86 |

Number of Ac-H4K12 immuno-positive cells (per 100 000 µm^2^) in ipsi- and contralateral hemisphere after MS-275 treatments. Student’s t-test did not reveal any difference between the hemispheres (all p’s > 0.05). aCSF n = 4; MS-275 500 µM n = 5; MS-275 1000 µM n = 3.
