# Supplementary material for: The Class I-Specific HDAC Inhibitor MS-275 Decreases Motivation to Consume Alcohol and Relapse in Heavy Drinking Rats
Source: Int J Neuropsychopharmacol. 2015 Apr 23;18(9):pyv029. doi: 10.1093/ijnp/pyv029 (PMC4576514; doi:10.1093/ijnp/pyv029)
Supplement: supplementary Figure S1 [file pyv029Supplementary_Figure_legends.doc]

Supplementary Figure S1. Schematic representation of the different areas used for the study of the MS-275 effect on H4 acetylation levels. Core, core of the nucleus accumbens; DLS, dorsolateral striatum; DMS, dorsomedial striatum, PFC, prefrontal cortex. The scale bar represents 100 µm.

Supplementary Figure S2. Profile of the extinction of the operant self-administration behavior over 13 sessions. The dotted lines represent the preextinction level (top one) and the extinction criterion of 20% (bottom one) of the baseline level.

Supplementary Figure S3. A single intra-cerebroventricular (i.c.v.) micro-injection does not alter ethanol self-administration on the day of the injection or the day after. Results are expressed as mean ± SEM number of active lever presses. n = 12.
