# Supplementary material for: The Class I-Specific HDAC Inhibitor MS-275 Decreases Motivation to Consume Alcohol and Relapse in Heavy Drinking Rats
Source: Int J Neuropsychopharmacol. 2015 Apr 23;18(9):pyv029. doi: 10.1093/ijnp/pyv029 (PMC4576514; doi:10.1093/ijnp/pyv029)
Supplement: supplementary Figure S1 [file Supplementary_Figures.pptx]

## Slide 1
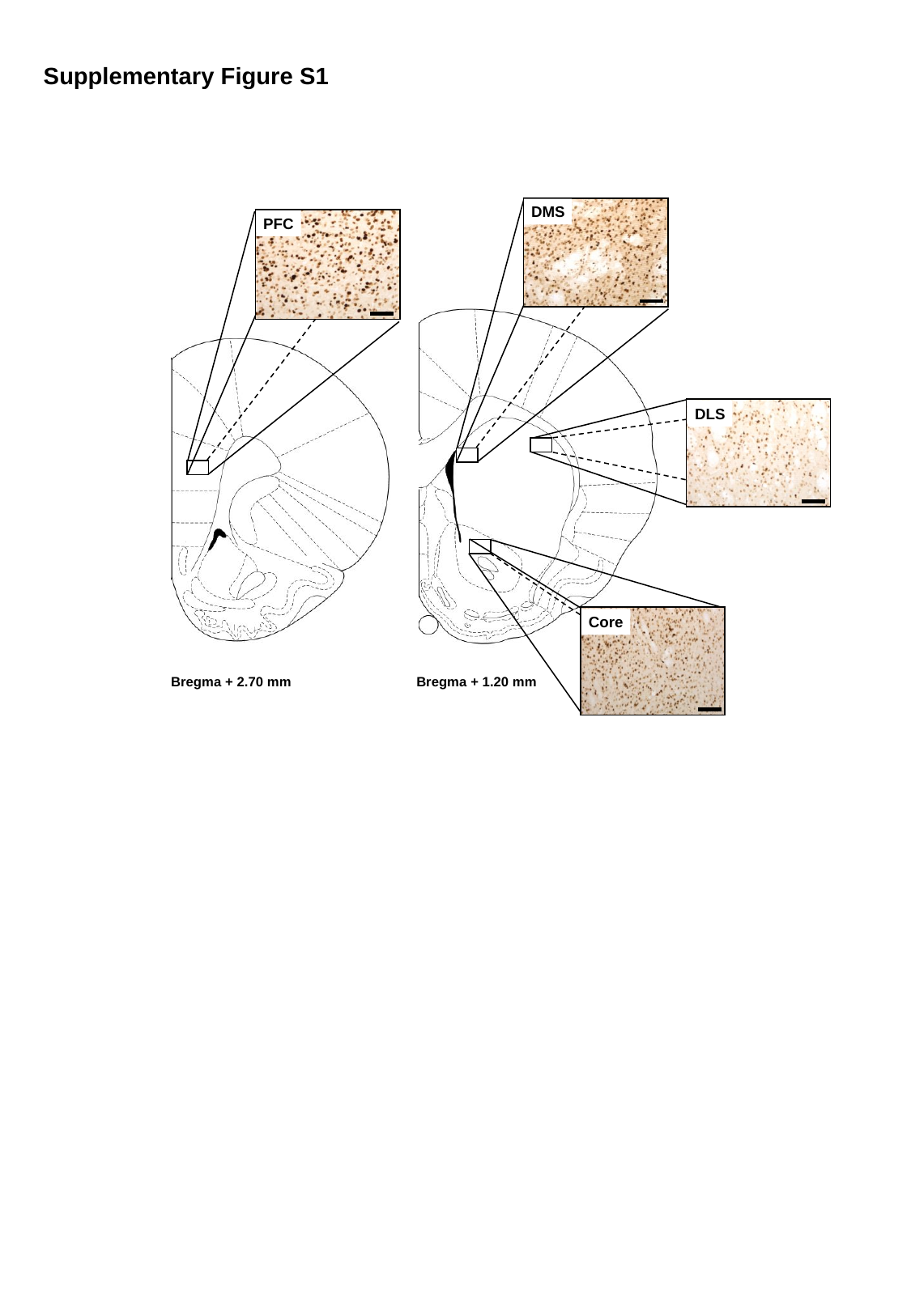

Supplementary Figure S1
DMS
PFC
DLS
Core
Bregma + 2.70 mm
Bregma + 1.20 mm

## Slide 2
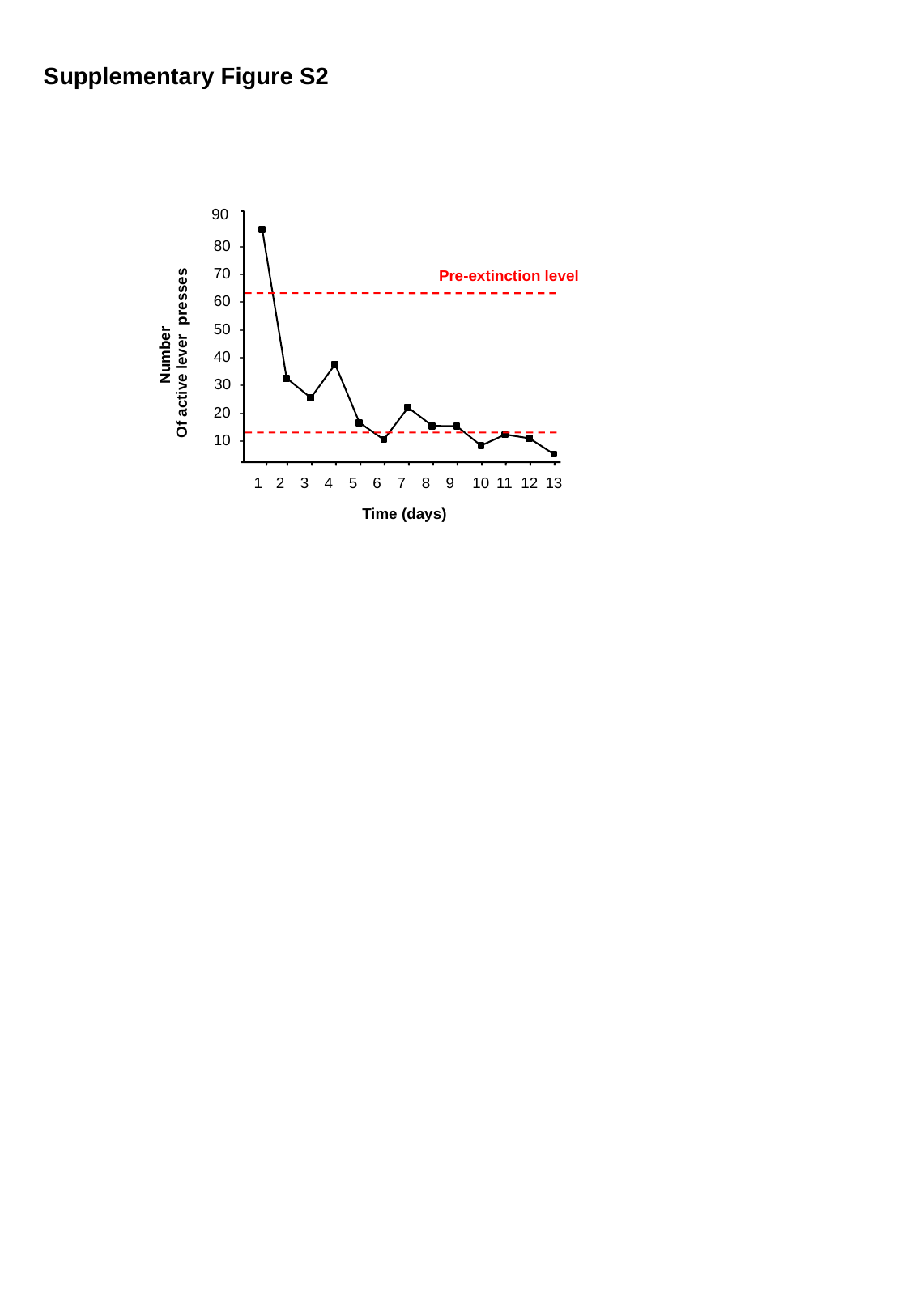

Supplementary Figure S2
90
80 -
70 -
Pre-extinction level
60 -
50 -
Number
Of active lever presses
40 -
30 -
20 -
10 -
1
2
3
4
5
6
7
8
9
10
11
12
13
Time (days)

## Slide 3
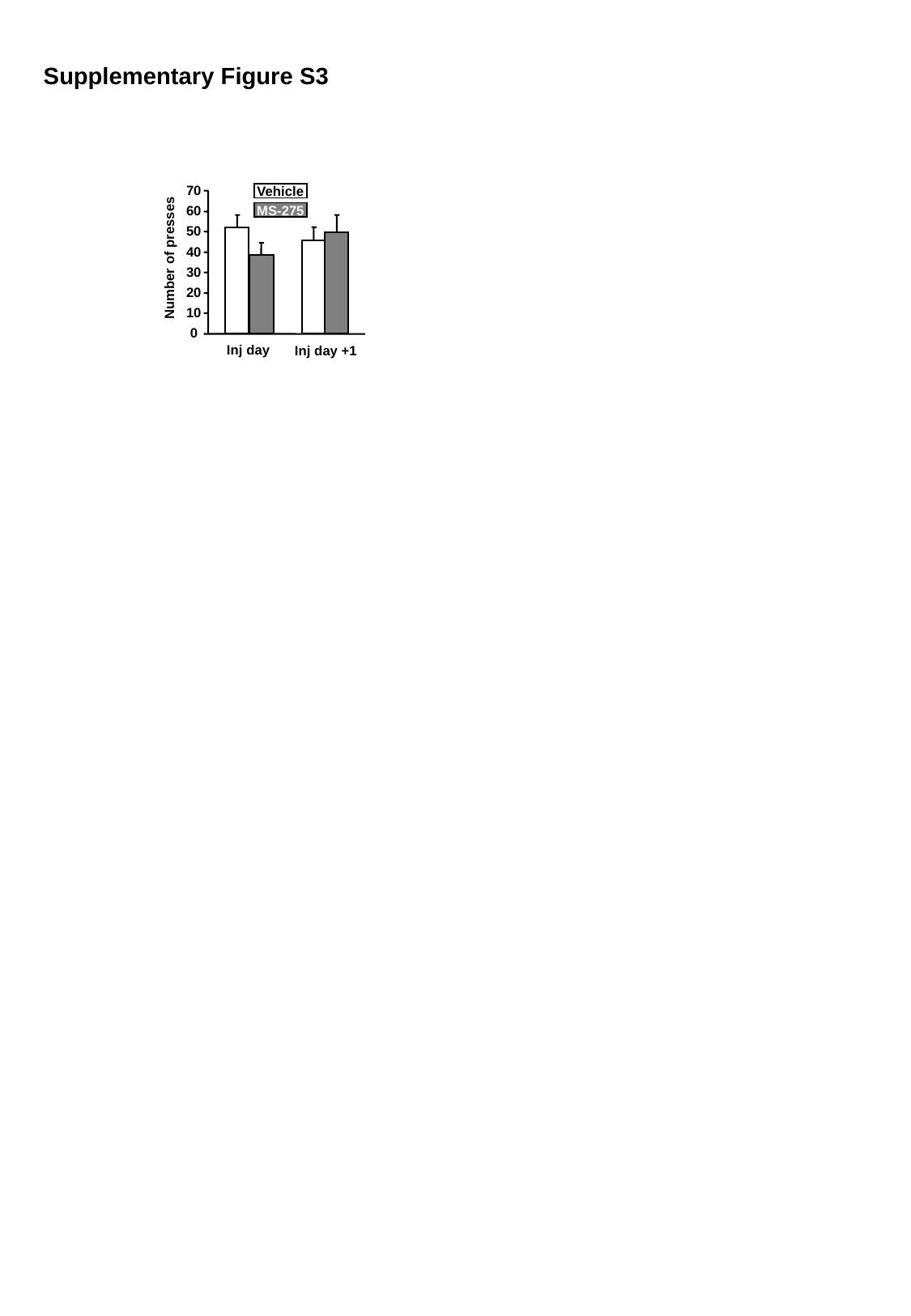

Supplementary Figure S3
70
60
50
40
Number of presses
30
20
10
0
Inj day
Inj day +1
Vehicle
MS-275
